# Age and work duration do not predict burnout in firefighters

**DOI:** 10.1186/s12889-019-6643-2

**Published:** 2019-03-14

**Authors:** Denis Vinnikov, Zhangir Tulekov, Alikhan Akylzhanov, Zhanna Romanova, Anar Dushpanova, Zhanna Kalmatayeva

**Affiliations:** 1 Al-Fabari Kazakh National University, al-Farabi avenue 71, Almaty, Kazakhstan 050040; 2 Biological institute, National Research Tomsk State University, Tomsk, Russian Federation 634050; 3 Fire Extinguishing and Rescue Service of Almaty City, Kunaev street 131, Almaty, Kazakhstan 050000

**Keywords:** Firefighter, Burnout, Alcohol, Logistic regression

## Abstract

**Background:**

The aim of this study was to ascertain the prevalence of burnout in Kazakhstan firefighters with regard to position and to identify predictors of faster burnout in order to plan future preventive strategies.

**Methods:**

Data on demographics, lifestyle, fatigue (Fatigue Severity Scale (FSS)), SF-8 health-related quality of life (HRQL) and Maslach Burnout Inventory (MBI) emotional exhaustion (EX), cynicism (CY) and professional efficacy (PE) were obtained from 604 (94% men, median age 27 (interquartile range (IQR) 12) years) firefighters from all 18 fire departments of the city of Almaty. Associations between predictors and burnout EX, CY and PE dimensions were tested using multivariate logistic regression analyses.

**Results:**

Burnout scores were low in this sample, including EX (0.6; IQR 1.55), CY (1.2; IQR 1.8) and PE (4.8; IQR 2.4). The highest median EX score (1.5 (IQR 2.0)) was in managers as opposed to the lowest in drivers (0.4 (IQR 1.4)), (*p* < 0.01). The greatest CY difference was between managers (2.1 (IQR 2.2)) and trainees (0.4 (IQR 1.1)) (*p* < 0.001). Age, work duration, education or fatigue were not associated with EX or CY in adjusted models. Better HRQL predicted lower EX and CY burnout, whereas alcohol never-use and language barrier predicted high CY. Male sex and no university degree predicted high PE burnout.

**Conclusions:**

Firefighting managers are at risk for higher burnout, irrespective of age and work duration, and the targeted intervention to combat burnout should include better uniform, mitigation of language barrier, general health improvement and less alcohol.

## Background

Occupational exposure to stress and high risk at work may be associated with faster burnout, also in those facing deadly accidents, such as firefighters. Burnout is a prolonged affective reaction to work stress, where an individual’s adaptive potential fails to meet work demands, which in high-risk occupations may be high. Independents of occupations, burnout is a consequence of exhaustion, and the overall concept of burnout assumes the interaction of the worker with the work environment. Exhaustion or fatigue usually results in burnout when an individual lack resources to combat growing gap between job demands and individual work capacity. Because burnout may provoke deteriorating professional efficacy, timely detection of accelerated burnout and its determinants may help plan and implement prevention in these high-risk occupations. Such determinants of faster burnout in firefighters have been poorly characterized, whereas the reports originate from a very few countries with quite small samples.

Available literature on burnout in firefighters concludes that job demands and work control may be associated with burnout, and the age of a firefighter may be associated with emotional exhaustion [1]. Some time later a study in American firefighters showed that work-family conflict may also provoke burnout [2], whereas another study added that neuroticism out of five selected personality traits had a positive correlation with firefighters’ burnout [3]. Such weariness at work will not only decrease professional efficacy, but may be associated with elevated injury rates [4], and given high physical activity level in this population, may lead to significant absenteeism. All these reports were, however, limited to small samples, and the issue whether these studies in French and American firefighters may be generalized to other countries, remains disputable.

No analysis has been performed from the post-Soviet countries to describe the level of burnout in such population, whereas the terms of reference and duties in firefighters may dramatically differ in these countries compared to the West. In Kazakhstan, firefighters are only involved in fight with fire at site with the least involvement of paramedic skills. We hypothesize that socioeconomic determinants may also affect burnout in Kazakh firefighters, because the occupation is in general poorly paid. With such poor compensation, we believe that work duration or even age may be positively associated with burnout, and little is known whether managing position within the firefighter system due to higher work demands can also predict weariness at work. Therefore, the aim of this study was to ascertain the prevalence of burnout in Kazakhstan firefighters with regard to position/rank and to identify predictors of faster burnout in order to plan future preventive strategies.

## Methods

The study was approved by the local Committee on Bioethics of the School of Public Health of al-Farabi Kazakh National University. For this analysis, all 18 fire department across the largest Kazakhstan city, Almaty (population around 2 million people) were asked to participate. The study was coordinated by the management of the Fire Extinguishing and Rescue Service of Almaty city, and all firefighters on active duty at a time of the study were included. Those on vacation, sick leave and business trip to other cities could not be enrolled. In the city of Almaty, each of 18 fire departments has four guards groups with two guard heads in each. The overall staff of departments differed depending on the area coverage. Each fire department has two persons on call, a manager with a vice, along with technicians and engineers.

We included from 12 to 50 firefighters from each department in this analysis. In Kazakhstan, a firefighter group includes several firefighters and a driver, who is not involved in active fight with fire, but is responsible for proper access and water supply. The sample also included engineers from the selected departments, driving instructors, managers, technicians, dispatchers, foremen and trainees. Each guard group is on duty for 24 h with the subsequent rest for 48 h. Moreover, firefighters in the country are all on military duty to warrant constant preparedness. In the current analysis, we grouped all subjects into 6 groups: 1 – drivers (*N* = 94); 2 – firefighters and senior firefighters (*N* = 242); 3 – firefighter trainees (interns) (*N* = 21); 4 – heads of the division and shift commanders (*N* = 130); 5 – department managers (*N* = 22) and 6 – other (*N* = 95). In total, we included 604 employees in this analysis.

We offered each subject a questionnaire which was divided into three sections, including the demographics part, health-related quality of life and fatigue scales, and finally questions on burnout. In the demographic part, we asked about the year of birth, sex, position, work duration in firefighting, marital status, highest attained education, self-reported smoking status with number of smoked cigarettes a day and smoking duration, alcohol consumption (never; sometimes in small amounts (less than once a week); sometimes in moderate amounts ((less than once a week); at least once a week), exercise at least 3 times a week for at least 40 min each and the use of physical activity tracker. In order to assess health-related quality of life, we used 8-item validated SF-8 general health-related quality of life tool and calculated the domains of physical activity, social activity, role physical, role emotional, mental health, vitality, physical pain and general health. We also calculated mental component score (MCS) and physical component score (PCS) as summative estimates of two domains of health-related quality of life.

Fatigue was measured with 9-item Fatigue Severity Scale (FSS), rating each item from 1 to 7 and summing all answers to produce FSS score. FSS score below or equal to 36 was treated as no fatigue, as opposed to score above 36 when fatigue was present. Burnout in firefighters was measured using Maslach Burnout Inventory (MBI) General (MBI-GS), consisting of 16 questions. This tool produces the average score for each of three dimensions, including emotional exhaustion (EX, 5 items, Cronbach α = 0.91), cynicism (CY, 5 items, α = 0.79) and professional efficacy (PE, 6 items, α = 0.82). EX scale measures feelings of being emotionally overextended and exhausted at one’s work, and the example is “I feel used up at the end of the workday”. CY scale measures an indifference or a distance attitude towards one’s work, and the example is “I have become less enthusiastic about my work”. Finally, PE scale measures feelings of competence and successful achievement in one’s work. The first two dimensions are expressed as a mean score of 5 questions and range from 0 to 5, whereas PE’s mean score ranges from 1 to 6. High mean score for the first two dimensions reflect high level of burnout. On the contrary, high scores of PE are indicative of low burnout for this dimension.

In addition, we also hypothesized that a number of other duty determinants may be associated with burnout and included three more questions. These were the questions whether a subject experienced meaningful carbon monoxide intoxication in the past, whether combat uniforms including duty boots wear entail any physical or psychological discomfort, and finally whether a subject experiences any language barrier at work in communicating with the colleagues. The latter was included because Kazakhstan is a bi-lingual country, where both Kazakh and Russian languages are almost equally present and used; however, older generations may be more fluent in Russian, whereas the newer generation born after the Soviet Union collapsed, may consider Kazakh as their first language.

The primary outcomes in this analysis were three dimensions of burnout, including EX, CY and PE, both as continuous and binary variables. We categorized the scores of each dimension into low-moderate and high reflecting three terciles of the overall range. Those falling into the third tercile (or above 66th percentile) were treated as having high level of burnout of each dimension. For univariate analyses, such as to compare groups, we used either contingency tables (for binary variables) or Student’s or Mann-Whitney U-test depending on distribution. Burnout dimensions, as most other variables in the current analysis were non-normally distributed with left-skewness; therefore we used non-parametric tests. Hence, data in the tables are expressed either as means with standard deviation or medians with the corresponding interquartile range (IQR). For each burnout dimension, categorized as high vs. low, we tested predictors, such as age, work duration, marital status, education, daily smoking, alcohol never use, exercising, SF-8 MCS and PCS, fatigue, language barrier, combat uniform wear discomfort and occupational group in the univariate analysis. Statistically significant predictors (*p* < 0.05) were then included in regression models, crude and adjusted for each other, thus treated as confounders. Such logistic regression produced odds ratios (OR) of each predictor or confounder with the corresponding 95% confidence intervals (CI). All tests were considered significant when *p* < 0.05. We used NCSS 12 (Utah, USA) for all calculations.

## Results

Of the overall sample of 604 included firefighters, 94% were men with notable left-skewness of work duration, reflecting low prevalence of experienced firefighters and high turnover rate (Table 1). Similarly, most subjects were young with strong left-skewness. Women differed from men in this sample almost in all demographic parameters, being older, working more years, attained higher education, but exercised less. In female firefighters, 60% had a university degree, reflecting their overall higher positions and greater likelihood of placement on a position of an engineer as opposed to a driver or firefighter in men. Smoking and alcohol use did not differ between male and female servicemen.

**Table 1:** The overall and sex-stratified summary of the sample

Indicator	All	Men	Women	p
N (%)	604 (100)	566 (94)	38 (6)	
Age, years	27 (12)	27 (11)	33.7 ± 6.5	0.001
Work duration, years	4 (9)	4 (9)	10.4 ± 6.1	0.002
Highest attained education, N (%)				
High school	205 (34)	199 (35)	6 (16)	0.001*
College	204 (34)	195 (34)	9 (24)	
University	195 (32)	172 (31)	23 (60)	
Marital status, N (%)				
Single	232 (38)	220 (39)	12 (31)	0.001*
Married	348 (58)	328 (58)	20 (53)	
Divorced	24 (4)	18 (3)	6 (16)	
Daily smokers, N (%)	228 (38)	222 (39)	6 (16)	0.01
Number of cigarettes a day^−1^	10 (10)	10 (10)	7.3 ± 4.5	0.20
Smoking duration, years	7 (8)	7 (7)	12.5 ± 9.4	0.35
Exercising at least 3 times a week, N (%)	277 (46)	272 (48)	5 (13)	0.001
Alcohol never-drinkers, N (%)	363 (60)	341 (60)	22 (58)	0.77

Note: * - data from a 2*3 table

**Fig. 1:**
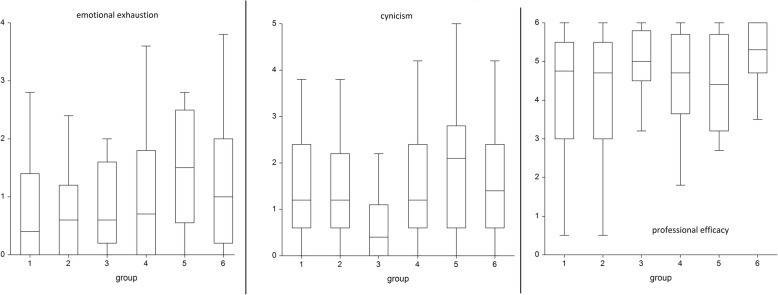
Emotional exhaustion, cynicism and professional efficacy in 6 groups of firefighters

In general, all three burnout dimensions were low in this sample, including EX (0.6; IQR 1.55), CY (1.2; IQR 1.8) and PE (4.8; IQR 2.4). There was no or very weak correlation between age and burnout or work duration and burnout dimensions. However, we found some significant differences in the burnout dimensions mean scores when groups were compared to each other, indicative of significantly greater burnout in department managers. The median EX scores for 6 groups were 0.4 (IQR 1.4); 0.6 (IQR 1.2); 0.6 (IQR 1.4); 0.7 (IQR 1.8); 1.5 (IQR 2.0); and 1 (IQR 1.8); therefore, the greatest difference was found between drivers and department managers (*p* < 0.01). The median CY scores for 6 groups were 1.2 (IQR 1.8); 1.2 (IQR 1.6); 0.4 (IQR 1.1); 1.2 (IQR 1.8); 2.1 (IQR 2.2); and 1.4 (IQR 1.8); therefore, the greatest difference was found between firefighter trainees and department managers (*p* < 0.001). The median PE scores for 6 groups were 4.8 (IQR 2.5); 4.7 (IQR 2.5); 5 (IQR 1.3); 4.7 (IQR 2.5); 4.4 (IQR 2.5); and 5.3 (IQR 1.3); however, none of the differences reached statistical significance. EX, CY and PE medians stratified by groups are shown in Fig. 1.

Table 2 demonstrates that a number of predictors were significantly different when subjects with high burnout were compared to their counterparts with low scores (below 66th percentile). With regard to EX, age and work duration could not predict high burnout score, as well as sex, smoking or regular exercising. Married firefighters, those with the university degree, poorer self-reported mental of physical health, high fatigue score, combat uniform discomfort and selected groups were more likely to show emotional burnout (Table 2). Table 2 shows that such predictors were not always similar for three burnout dimensions; and with regard to PE, those were only sex, university degree and a position group.

**Table 2:** Univariate analysis of burnout dimensions

Predictor	EX		CY		PE	
	High (*N* = 181)	Low (*N* = 423)	High (*N* = 201)	Low (*N* = 403)	High (*N* = 198)	Low (*N* = 406)
Age	28 (14)	27 (11)	28 (14)	27 (11)	28 (13)	27 (12)
Male sex, N (%)	165 (91)	399 (94)	187 (93)	377 (94)	193 (97)	371 (91)*
Work duration, years	5 (11)	4 (9)	5 (10)	4 (9)	4 (9)	4 (9)
Group 1	24 (13)	69 (16)	29 (14)	64 (16)	38 (19)	55 (14)
Group 2	56 (31)	186 (44)*	75 (37)	167 (41)	94 (47)	148 (36)*
Group 3	6 (3)	15 (4)	1 (0)	20 (5)*	2 (1)	19 (5)*
Group 4	44 (24)	86 (20)	46 (23)	84 (21)	40 (20)	90 (22)
Group 5	12 (7)	9 (2)*	14 (7)	7 (2)*	8 (4)	13 (3)
Group 6	39 (22)	58 (14)*	36 (19)	61 (15)	16 (8)	81 (20)*
Married, N (%)	115 (64)	231 (55)*	116 (58)	230 (57)	113 (57)	233 (57)
University education	71 (39)	123 (29)*	76 (38)	118 (29)*	47 (24)	147 (36)*
Daily smoking	71 (39)	157 (37)	85 (42)	143 (35)	76 (38)	152 (37)
Exercising 3 times a week	78 (43)	199 (47)	86 (43)	191 (48)	85 (43)	192 (47)
Alcohol never use	90 (50)	271 (64)*	98 (49)	263 (65)*	125 (63)	236 (58)
SF-8 PCS	61.2 (9.4)	64.8 (4.1)*	62.3 (7.0)	64.8 (4.4)*	64.2 (6.0)	64.8 (5.2)
SF-8 MCS	60.9 (11.6)	66.3 (5.7)*	62.4 (10.4)	66.2 (5.9)*	64.8 (7.3)	65.1 (8.0)
FSS > 36	25 (14)	22 (5)*	24 (12)	23 (6)*	14 (7)	33 (8)
Language barrier	17 (9)	24 (6)	26 (13)	15 (4)*	9 (5)	32 (8)
Combat uniform wear discomfort	52 (29)	44 (10)*	46 (23)	50 (12)*	31 (16)	65 (16)

Note: *EX* emotional exhaustion, *CY* cynicism, *PE* professional efficacy, *MCS* Mental Component Score, *PCS* Physical Component Score, *FSS* Fatigue Severity Scale; * - *p* < 0.05

When significant univariate analysis predictors were included in adjusted regression models, we found that predicting role of most significant predictors was mediated though other, since in adjusted models they could no more predict burnout, including marital status or university degree as an example. As Table 3 shows, in adjusted models only poorer self-reported health-related quality of life (SF-8 PCS and MCS) and combat uniform wear discomfort could predict higher EX burnout. With regard to CY, the list of predictors was wider, and alcohol use, self-reported health-related quality of life and language barrier predicted higher burnout, when the latter increased the odds of high burnout almost 3-fold. Finally, high PE burnout was associated with male sex (OR 3.40; 95% CI 1.17–9.91) and lower educational level.

**Table 3:** Regression analysis of burnout predictors

	EX		CY		PE	
Sex	–	–	–	–	4.43 (1.55–12.7)	3.40 (1.17–9.91)
Group	2.93 (1.24–6.92)	1.92 (0.67–5.45)	3.70 (1.52–8.96)	2.66 (0.98–7.20)	1.58 (1.12–2.22)	1.34 (0.94–1.91)
Marriage	0.67 (0.47–0.97)	0.88 (0.58–1.33)	–	–	–	–
University degree	1.56 (1.08–2.24)	1.27 (0.83–1.94)	1.45 (1.01–2.07)	1.13 (0.76–1.68)	0.54 (0.37–0.80)	0.61 (0.41–0.90)
Alcohol never	0.56 (0.39–0.80)	0.84 (0.56–1.26)	0.51 (0.36–0.72)	0.65 (0.45–0.94)	–	–
SF-8 MCS	0.90 (0.87–0.92)	0.93 (0.90–0.96)	0.92 (0.89–0.95)	0.95 (0.92–0.98)	–	–
SF-8 PCS	0.87 (0.84–0.91)	0.90 (0.87–0.93)	0.92 (0.89–0.95)	0.95 (0.92–0.98)	–	–
Fatigue	2.92 (1.60–5.33)	1.66 (0.84–3.37)	2.24 (1.23–4.08)	1.40 (0.71–2.73)	–	–
Combat uniform discomfort	3.60 (2.28–5.58)	2.60 (1.58–4.29)	2.15 (1.39–3.35)	1.38 (0.85–2.26)	–	–
Language barrier	–	–	3.84 (1.99–7.45)	2.77 (1.35–5.65)	–	–

Note: *EX* emotional exhaustion, *CY* cynicism, *PE* professional efficacy; adjusted ORs are adjusted for all predictors in a model; group analysis – group 5 vs. the rest in EX, group 5 vs. the rest in CY, group 2 vs. the rest in PE

## Discussion

To our knowledge, this is the largest analysis of burnout in firefighters, which also includes regression models of selected predictors of weariness at work. We found that the overall prevalence in firefighters was quite low. Neither work duration, nor age, but poorer health-related quality of life and combat uniform discomfort were associated with higher EX burnout; alcohol use, language barrier and poorer health-related quality of life could predict higher CY burnout, whereas male sex and lower education were indicative of burnout in PE. We also found that, irrespective of work duration, managers in fire fighting had higher burnout in EX and CY scales compared to other groups. We consider these findings somewhat surprising, but new, because they shed light to previously understudied determinants of burnout, thus guiding future directions for its prevention.

Position within firefighting system was associated with selected burnout dimensions. Managers had higher levels of burnout in EX and CY, whereas regular firefighters had higher scores of professional efficacy burnout. Managing position in firefighting is assumed to have higher job demands compared to firefighters, therefore, managers are more likely to be emotionally exhausted. Such an association of higher work demands with greater EX was confirmed in a previous study [1], but no stratification into positions was done there. A recent nationwide study from Germany confirmed the association of high demands with burnout, greater in women and independent of individual predictors [5]. Our study now adds evidence to such an association with clarification that higher job demands as a surrogate of higher position within the system may provoke accelerated burnout. Of note, no position-stratified burnout was ever studied in firefighters before, whereas the scarce literature on burnout in managers is mostly based on studies in healthcare personnel.

Our analysis showed that marital status, education, alcohol use and fatigue were associated with selected dimensions of burnout not directly, but the effect was likely mediated through other predictors. Health-related quality of life in this study was associated with both EX and CY, which may be distinctly plausible, however, we could not find other studies referring to health-related quality of life in firefighters for comparison. Moreover, we assumed in the beginning that fatigue should explain some variability in burnout, but in adjusted models it was also found to have non-significant associations. We also find it hard to compare the overall burnout scores with other studies in firefighters, because in those few studies available they used different tools of burnout producing various scales. Nevertheless, some similar associations of predictors with burnout were identified in other occupational studies. Thus, alcohol use was shown to have a positive association with burnout in intensive care physicians [6], community pharmacists [7], prison employees [8], priests [9], and similar association was found in a populations-based sample [10]. Since data on firefighters on such association were not published before, findings from the latter population-based study assume that such association is not specific for firefighters and can be found in many other occupations.

One of the novel findings in firefighters was the association of combat uniform wear discomfort with EX, and language barrier with CY. In the published literature we could not identify other studies quantifying the association of combat wear discomfort with burnout, and such association may have a previously understudied psychological underpinning. Independent of the mechanism, this has a clear implication for the fire department management to mitigate burnout with better uniforms or boots to satisfy fighters’ needs for comfort physical and psychological conditions. With regard to language barrier as a predictor of cynicism, possible psychological weariness mechanism is plausible. Again, clear implications for practice arise from this association, and firefighting management should address the issue of strong bilingualism in the personnel in order to slow down burnout cynicism.

Although some association of sex with selected dimensions of burnout was reported in other studies with non-firefighting occupations, it is likely gender-specific as opposed to biological sex-associated phenomenon, since no difference in neurotransmitters were found in men compared to women with regard to emotional exhaustion [11]. Our findings exhibit greater male vulnerability for PE burnout, even adjusted for educational level, and university degree was protective against this burnout domain.

Our study has some limitations. Firstly, we could only collect data from Almaty, though the largest city in the country. Kazakhstan has a large territory, and cities in the North may have specific organizational matters, which could affect burnout. Secondly, we could not verify the direction of emerged association because of the cross-sectional study design. Thus, at present we cannot say whether discomfort from the combat uniform predicts emotional exhaustion or vice versa. Same relates to other dimension of burnout in this study. Moreover, there likely existed other unmeasured confounding on top of the proposed variables, such as language barrier or carbon monoxide intoxication in the past or combat uniform discomfort. Finally, age left-skewness in the studies sample probably resulted in the overall low burnout in all dimensions as a result of some selection bias, because older and likely more exhausted at work fighters had lower likelihood of being studied.

This study has distinct polity implications for the firefighting management to reduce burnout. Our initial hypothesis of the association of working duration and age in such high-risk occupation could not be confirmed, and instead we found that efforts should rather be directed to improve communication skills in the department to reduce burnout cynicism and improve combat uniform wear comfort to ameliorate emotional exhaustion. Firefighters should also be offered other coping strategies to replace alcohol use to lessen cynicism. Fire department managers, likely because of greater job demands, may be more prone to burnout; therefore, prevention should be targeted to these groups. Similarly, regular firefighters may exhibit greater burnout in professional efficacy; hence, training, retraining and encouragement may be offered to them, but the effect of such interventions should be tested in well-planned studies.

## Conclusions

Burnout as measured with emotional exhaustion, cynicism and professional efficacy in Kazakhstan firefighters is low, but higher in management compared to other positions. Age and work duration may not predict any of the studied three dimensions of burnout. There is a need for targeted burnout mitigation programs based on the identified predictors for selected dimensions.

## Abbreviations

CI: Confidence interval
CY: Cynicism
EX: Emotional exhaustion
FSS: Fatigue severity scale
HRQL: SF-8 health-related quality of life
IQR: Interquartile range
MBI: Maslach Burnout Inventory
MCS: Mental component score
OR: Odds ratio
PCS: Physical component score
PE: Professional efficacy
